# Sampling in health geography: reconciling geographical objectives and probabilistic methods. An example of a health survey in Vientiane (Lao PDR)

**DOI:** 10.1186/1742-7622-4-6

**Published:** 2007-06-01

**Authors:** Julie Vallée, Marc Souris, Florence Fournet, Audrey Bochaton, Virginie Mobillion, Karine Peyronnie, Gérard Salem

**Affiliations:** 1 Conditions et Territoires d'Emergence des Maladies (UR178), Institut de Recherche pour le Développement (IRD), UR 178, PO. 5992, Vientiane, Laos; 2 Laboratoire Espace, Santé et Territoire, Université Paris X- Nanterre, 200 avenue de la République, 92000 Nanterre, France; 3 Conditions et Territoires d'Emergence des Maladies (UR178), Institut de Recherche pour le Développement (IRD), Mahidol University, Bangkok, Thailand; 4 Conditions et Territoires d'Emergence des Maladies (UR 178), Institut de Recherche pour le Développement, BP 182, Ouagadougou, Burkina Faso

## Abstract

**Background:**

Geographical objectives and probabilistic methods are difficult to reconcile in a unique health survey. Probabilistic methods focus on individuals to provide estimates of a variable's prevalence with a certain precision, while geographical approaches emphasise the selection of specific areas to study interactions between spatial characteristics and health outcomes. A sample selected from a small number of specific areas creates statistical challenges: the observations are not independent at the local level, and this results in poor statistical validity at the global level. Therefore, it is difficult to construct a sample that is appropriate for both geographical and probability methods.

**Methods:**

We used a two-stage selection procedure with a first non-random stage of selection of clusters. Instead of randomly selecting clusters, we deliberately chose a group of clusters, which as a whole would contain all the variation in health measures in the population. As there was no health information available before the survey, we selected *a priori *determinants that can influence the spatial homogeneity of the health characteristics. This method yields a distribution of variables in the sample that closely resembles that in the overall population, something that cannot be guaranteed with randomly-selected clusters, especially if the number of selected clusters is small. In this way, we were able to survey specific areas while minimising design effects and maximising statistical precision.

**Application:**

We applied this strategy in a health survey carried out in Vientiane, Lao People's Democratic Republic. We selected well-known health determinants with unequal spatial distribution within the city: nationality and literacy. We deliberately selected a combination of clusters whose distribution of nationality and literacy is similar to the distribution in the general population.

**Conclusion:**

This paper describes the conceptual reasoning behind the construction of the survey sample and shows that it can be advantageous to choose clusters using reasoned hypotheses, based on both probability and geographical approaches, in contrast to a conventional, random cluster selection strategy.

## Background

Geography is an independent scientific approach, while statistics simply constitutes a body of methods and tools that can be employed by scientists from various fields of research. Probabilistic statistics can be used to estimate a variable's prevalence in a given population with a certain precision. However, analysing survey data in this way can become complicated when the sample is selected using a geographical approach. The geographical approach often favours the study of specific areas, deliberately chosen to enable analysis of processes and interactions that are key to the understanding of particular health behaviours and spatial disparities. Cluster samples chosen from a small number of specific areas raise an important statistical challenge, in that the non-independence of observations within clusters has an impact on the statistical validity of the sample at the global level. Waldo Tobler showed in his first law of geography that "everything is related to everything else, but near things are more related than far things" [1]. Therefore, statistical methods for analysing spatial data have to take into consideration spatial arrangements, and the resulting correlations between observations, in order to provide accurate and meaningful conclusions [2]. This article suggests a method for the reconciliation of probabilistic statistical methods and geographical objectives in a unique health survey in Vientiane, the capital of Lao People's Democratic Republic (Lao PDR). In this method, areas are selected so that respondents are as representative of the general population as possible, whilst still enabling the study of health spatial interactions at the local level.

## Problem statement

### A need for meaningful data for public health and for health geography

The geographical approach calls for selecting specific places from where health information about large segments of the population can be acquired, in order to study interactions between people living in the same place. Selection of specific areas allows precise descriptions of the environment (e.g., the ecological landscape, medical equipment availability, markets, relevant policies, and so on) and its relationship with health, which would be very difficult to assess for a whole city. It is interesting to select some relevant territories where it is possible to study health spatial disparities, to explore interactions between people and places and to gain a better understanding of spatial organisation in a given society. Additionally, to analyse health spatial disparities, geography researchers often distinguish between the effects of "context" (e.g., area or group properties) and "composition" (characteristics of individuals living in different areas) in contextual and multilevelanalyses; these analyses therefore require datasets including individuals nested within areas or neighbourhoods [3]. In conclusion, to conduct geographical analyses (spatial interaction, spatial correlation, contextual and multilevel analysis), researchers need to carry out a health survey in specific places where people and their neighbours can be interviewed.

At the same time, it would be useful if the study also produced an estimate of prevalence for each important health variable, and identified health-seeking behaviours and individual risk factors. Such findings help inform public health policy decisions. Indeed, as the Asian Development Bank noted regarding Lao PDR, "there is an urgent need for a nationwide survey of household sanitation in urban areas. This can be a sample survey as long as the sample size is representative" [4]. Therefore, the results of a survey, even one in which data are collected in specific areas, must also be representative for the whole area.

Stratification can improve the representativeness of a sample by reducing sampling errors, and can make variance estimates more precise [5]. If surveyed individuals were chosen in every stratum by simple random sampling, we would have achieved a good representative sample of the stratum population; however, if this is done, geographical approaches that examine how individuals interact with their wider environment are not possible [6]. In a health survey recently carried out in Lao PDR, we needed to define and select some relevant territories where it was possible to study health spatial disparities, to explore interactions between people and places and to gain a better understanding of spatial organisation in a given society.

### Conventional random cluster selection

#### Design effects

The most commonly used spatial sampling method is cluster sampling: the studied area is divided into units, and a selection is then randomly chosen. Within each unit, individuals are ideally chosen by simple random sampling. Cluster sampling economises on time, budgets and energy; it is often done primarily for these practical reasons, as in the Expanded Programme on Immunisation (EPI) [7], because it is less expensive than simple random sampling when the population is dispersed. Cluster designs can also be useful in geographical approaches, because they allow for the study of specific places and territories. However, such designs are often less precise than simple random sampling, due to the homogeneity of individuals within clusters. There may be good reasons why individuals' behaviour within a small area is similar: "Why should we expect independence in spatial observations (...)? All our efforts to understand spatial patterns, structure and process have indicated the lack of independence (...) of things in time and space" [8]. With cluster sampling, every member of the population has an equal chance of selection, but individuals with similar characteristics are more likely to be surveyed. Cluster sampling necessarily has a design effect, making it less statistically robust than simple random sampling [5]. This effect varies between areas and even within the same area, and it can vary depending on the question. The size of the design effect can be calculated after the study as the variance obtained from the cluster sample divided by the variance that would have been obtained with a simple random sample of equal number.

#### Number of selected clusters

The statistical precision of cluster sampling can be improved by increasing the number of clusters. At the extreme, when all of the clusters are sampled, one effectively has a stratified sample, typically associated with increased levels of precision. At the other extreme, if only one cluster is selected, results would be specific to that cluster and not necessarily generalizable to the global population. To improve representativeness of the overall sample, it is best to select many areas, even if this means sampling fewer individuals within each area [9]. It is often hard to find the best compromise between the number of clusters and the number of individuals per cluster. It is interesting both to study the heterogeneity of health characteristics between different areas and to study the homogeneity of characteristics in the same area. The ideal approach would be to survey a large population in a large number of areas. However, when budgetary constraints must be taken into account, one must often choose the number of clusters according to the following criteria:

1. The number of clusters must be sufficiently large that statistical precision at the population level is adequate, and spatial comparisons remain possible, and

2. The number of clusters must be sufficiently small for the survey to remain logistically feasible, and for geographical analyses to be performed properly at the local scale.

### The argument against random selection of clusters

Random cluster selection does not necessarily mean that the sample of clusters is representative of the whole population, especially when the number of clusters sampled is small; in fact, the design effect is highly dependent on this number. The variance obtained by cluster sampling can be reduced by selecting areas that are as internally heterogeneous (i.e. have a full range of variability within them) as possible and as externally homogeneous (i.e. are as similar to one another) as possible. The ideal situation, in terms of statistical precision, would be that each area was a microcosm of the entire population and, therefore, was perfectly representative, in terms of variability, of the overall population [9]. However, this solution is in opposition to geographical objectives, in that the geographic approach (as in the spatial statistics approach) views population homogeneity as interesting in itself. Herein lies the difficulty: the design effect could be reduced by choosing similar areas with high internal heterogeneity, but other than the fact that it is impossible in practice to identify such an area, this would be in contradiction with the objectives of health geography.

To reconcile probabilistic methods and geographical objectives, we propose purposeful, rather than random, selection of a group of clusters that together could contain all the variability of the overall population.

### How do we select clusters to best reproduce the population distribution of variables?

To improve the reliability of our sample, consisting of a small number of clusters, we have to choose the best combination of *n *clusters, such that respondents are representative of population heterogeneity (with regards to, in this case, health variables). Since no health information is available before the survey, a *priori *variables that can influence the spatial homogeneity of health characteristics in the studied area have to be determined. The notion of resemblance is subjective, but it aims to ensure that any two given populations resemble each other in terms of the phenomenon being researched. The choice of *n *clusters is thus based on reasoned hypotheses and on the specific research objectives. Cluster selection is therefore conceptually derived from a set of definite hypotheses, which is necessarily different from the hypotheses another group of researchers might have. Among every available variable, we select well-known health determinants (e.g. age, nationality, ethnic origin, education, occupation, etc.) and keep some variables (v_1_; v_2_...v_n_) with unequal spatial distribution within the studied area. It is possible to check the survey results after the research has been carried out for *a posteriori *relevance of the given variables as health determinants.

To select the combination of *n *clusters whose composition is most similar to the composition of the overall studied area, it is first necessary to list all the possible combinations of *n *clusters (without repetition) among *N *clusters existing in the studied area. Usually, we obtain a large number of combinations:

Cb=CnN=N!n!×(N−n)!
 MathType@MTEF@5@5@+=feaafiart1ev1aaatCvAUfKttLearuWrP9MDH5MBPbIqV92AaeXatLxBI9gBaebbnrfifHhDYfgasaacH8akY=wiFfYdH8Gipec8Eeeu0xXdbba9frFj0=OqFfea0dXdd9vqai=hGuQ8kuc9pgc9s8qqaq=dirpe0xb9q8qiLsFr0=vr0=vr0dc8meaabaqaciaacaGaaeqabaqabeGadaaakeaacqWGdbWqcqWGIbGycqGH9aqpcqWGdbWqdaqhaaWcbaGaemOBa4gabaGaemOta4eaaOGaeyypa0ZaaSaaaeaacqWGobGtcqGGHaqiaeaacqWGUbGBcqGGHaqicqGHxdaTcqGGOaakcqWGobGtcqGHsislcqWGUbGBcqGGPaqkcqGGHaqiaaaaaa@411C@

Where *Cb *is the number of possible combinations of *n *clusters without repetition; *N *is the total number of clusters existing in the studied area and *n *is the number of clusters we want to select.

It is then necessary to calculate, for each combination of *n *clusters, the mean squared difference between the proportion of v_1 _(we call it Pv_1_) in each of the *n *clusters (c_1_; c_2_...*c*_n_) and the mean proportion of v_1 _calculated at the studied area level (Pv_1area_).

*Mean squared difference *= [(Pv_1 _c_1- _Pv_1area_)^2 ^+ (Pv_1 _c_2- _Pv_1area_)^2 ^+ (Pv_1 _c_3- _Pv_1area_)^2 ^+...+ (Pv_1 _c_n- _Pv_1area_)^2 ^]/n

This mean squared difference is compared with the variance of v_1 _calculated at the studied area level. Cluster combinations are retained where the mean difference corresponds with the variance calculated for the studied area. The same steps are followed with every selected variable (v_2_...v_n_). Among the large number of possible combinations *of n *clusters, few combinations are obtained, whose variability of different selected variables is very similar to the variability calculated at the studied area level. This procedure enables clusters to be selected that have a composition of *a priori *health determinants that is similar to the composition of the *a priori *health determinant in the overall studied area. With this procedure, we hope to reduce design effects and gain statistical precision while surveying only a few clusters.

## Applications in Vientiane health survey

We applied this form of clustered sampling to a health survey conducted in Vientiane, Lao PDR.

### Health survey in Vientiane

The main objective of the research programme, entitled "Urbanization, Governance and Spatial Disparities of Health in Vientiane", is to describe and analyse the organisation of urban areas (including geographical, social, cultural, political, environmental, and behavioural variables) as sources of intra-urban health inequalities. The urbanised area of Vientiane is spread over 148 villages ('ban' in Lao) comprising approximately 277,000 inhabitants in 2005 [10]. In Lao PDR, the "village" (*ban*) is the smallest administrative, religious and political unit in both rural and urban areas. The spatial division into villages reflects political, administrative and social reality and, as census data are available at the village level, we decided to keep the village as the reference unit for the survey: a cluster thus corresponds to a village. In 2005, an average of approximately 1870 people lived in a Vientiane City village (interquartile range: 1080 – 2311). It is likely that the increased urbanisation of the capital has led to wide disparities in health, but as little health information exists, there is no way to know what kind of health problems the population encounters and how people seek healthcare. To provide health data and to analyse health spatial disparities in Vientiane, the French Research Institute for Development (Institut de Recherche pour le Développement – IRD) carried out a health survey within the city in collaboration with the Lao Ministry of Health, the National Institute of Public Health, the Faculty of Medical Sciences, the Francophone Institute of Tropical Medicine (Institut Francophone de Médecine Tropicale – IFMT) and the Microbiology Laboratory in Mahosot Hospital. Ethical approval for this survey was obtained from the Lao National Ethics Committee for Health Research in Lao PDR.

Two age groups were selected: children (aged from six months to less than six years) and adults (aged 35 years and above). Data were collected in February and March 2006 through household and individual questionnaires. Household questionnaires collected data on house location and description, living conditions, incomes, community bonds and demographic data on every member of the household. Individual questionnaires collected demographic data and socio-economic information, urban lifestyle variables, behavioural risk factors, health status data, and healthcare-seeking behaviours. Health status was measured through medical examination and investigations (weight, height, temperature, blood pressure, dental examination and blood samples from a fingerprick to study diabetes, anaemia and communicable diseases). Healthcare-seeking behaviour was examined through questions about type and gravity of health problem, local health structure, price, quality, and satisfaction with health care services.

With these data, we aimed to: (i) compare levels of morbidity in different urban areas; (ii) identify appropriate urban scales for recognising health disparities; (iii) detect hotspots of morbidity using exploratory spatial data analysis; and (iv) measure the impacts of both social and urban contexts using multilevel analyses.

### Use of urban stratification

**Figure 1:**
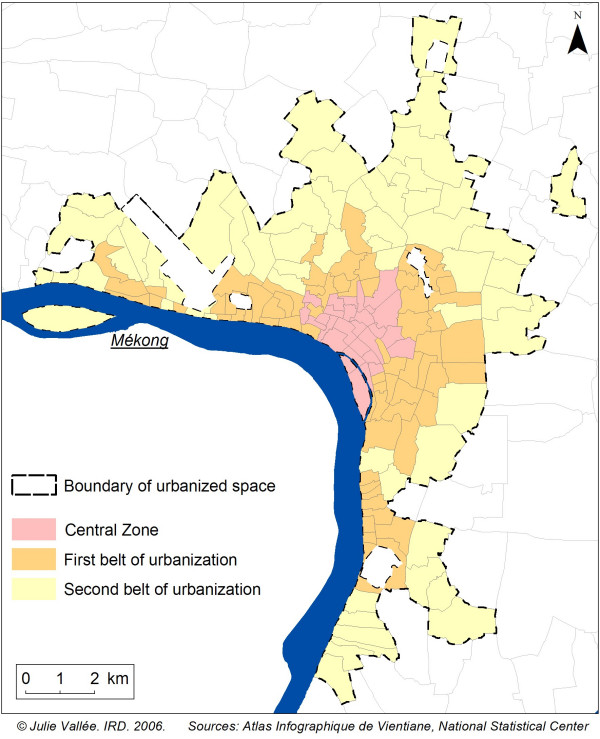
**Urban stratification in Vientiane**. 3 areas of decreasing degree of urbanization can be distinguished in Vientiane: a central zone, a first belt and a second belt of urbanization.

As the main objective of this programme was to study the relationship between urbanisation and health, the first stage of this health survey required us to set the spatial limits of the urbanised area of Vientiane and to stratify this defined area [10]. To set the limits of an urban area, it was more relevant to use a variety of census-based and GIS-based indicators rather than a single common indicator such as population density. We selected 13 different indicators describing urbanisation in the urban area of Vientiane: the proportion of built-up area; density of the population; changes in the built-up surface area between 1981 and 1999; the proportion of public infrastructure buildings; the proportion of trade buildings; the number of markets nearby; distance to the city centre via the road network; the average distance of every building to the road network; access to running water, electricity and toilets; the proportion of concrete houses; and the proportion of the population involved with agricultural activities. These indicators are derived from the 1999 aerial photographs processed in "Atlas Infographique de Vientiane" [11] and the 1995 census from the Lao National Statistical Center. Using a hierarchical classification, we differentiated Vientiane's villages into three strata of decreasing level of urbanisation (central zone, first urbanised belt and second urbanised belt) with 25, 67 and 56 villages in each stratum respectively (Figure 1) [10]. The proportions of the population living in these three strata in 2005 are unequal: 11.7% in the central zone, 40.7% in the first urbanised belt and 47.7% in the second belt. It was therefore important to stratify by degree of urbanisation if we wished to survey a sufficient number of individuals in each stratum, especially in the central zone where the population was smallest.

### Number of villages to survey

Budgetary limitations affected the overall sampling size: 2000 adults and 2000 children for the whole city (or 666 adults and 666 children in each urban stratum). This allowed for a 95% CI of +/- 2.3% around a prevalence of 10% at the stratum scale, and +/-1.3% around a prevalence of 10% at the city scale. In this calculation, we have not considered the design effect. The value of the design effect (which differs between variables within the same survey) is difficult to estimate during survey preparation, and is very dependent on the selection of clusters. We planned to survey the same number of individuals in every village so that comparison could be done with the same precision. According to the size of the village population, we fixed a sample size of 27 villages (nine per urban stratum), with 74 adults and 74 children to be sampled in each village. This corresponds to a mean sampling rate of 1/5.6 for adults and 1/2.4 for children, based on the list of households created in December 2005 in every one of the 27 selected villages.

### Selection of villages

For logistical convenience, we decided to re-group these 27 villages into nine groups of three adjacent villages, such that only one medical centre was needed for every three adjacent villages. Using a contiguity matrix, we listed in each stratum all the possible groups of three adjacent villages and produced all the combinations of three groups of three adjacent villages without repetition. We obtained a very large number of combinations (table 1), from which we selected the combination of nine villages that was most representative of the stratum in both urban and health terms.

**Table 1:** Number of combinations in every urban stratum

	Central zone	First urbanised belt	Second urbanised belt
Number of villages	25	67	56
Number of possible groups of 3 adjacent villages	102	209	92
Number of possible combinations of 3 groups of 3 adjacent villages	46 828	999 140	60 225
Number of possible combinations of 3 groups of 3 adjacent villages which respect proportions of urbanization types.	6 819	10 851	2 408

#### Urban representativeness

**Figure 2:**
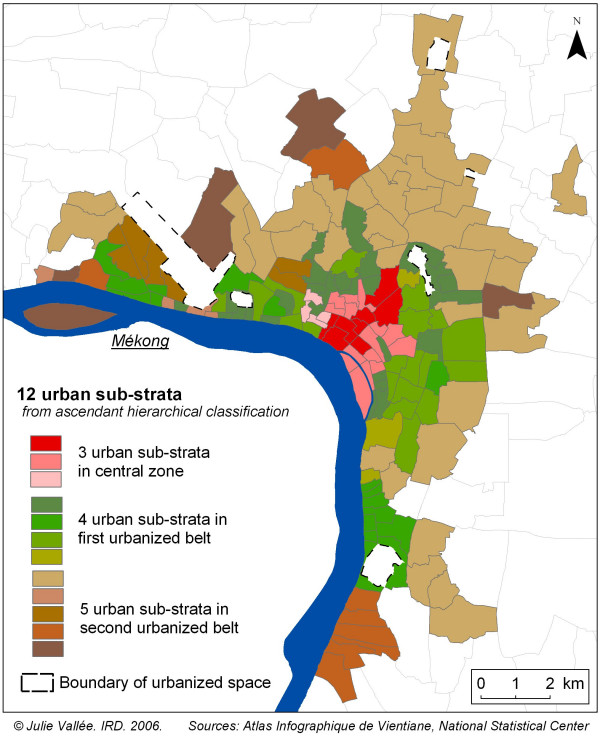
12 urban sub-strata in Vientiane: 3 sub-strata in the central zone, 4 in the first urbanised belt and 5 in the second urbanised belt.

Among the combinations of three groups of three adjacent villages, we pre-selected combinations that were representative of urban characteristics. Despite already having stratified on degree of urbanisation, the small number of studied villages (nine) in each stratum still did not guarantee a good representation of urban characteristics within the stratum itself. To solve this problem, we used our hierarchical classification of urban characteristics to distinguish further sub-strata of urbanisation within each urban stratum, and defined three substrata in the central zone, four in the first urbanised belt and five in the second urbanised belt (Figure 2). For each possible combination of nine villages, we calculated the proportions of villages belonging to the different urban sub-strata, and the mean of differences between these proportions and those for the whole stratum. We pre-selected every combination of nine villages for which the mean of the differences was lower than 5% (table 1).

#### Health representativeness

**Figure 3:**
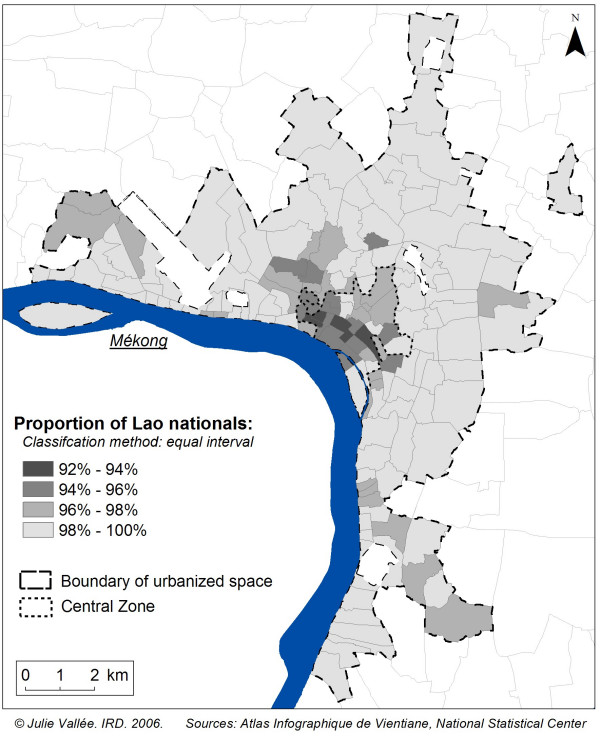
**Spatial distribution of proportion with Lao nationality in 1995**. The majority of non-Lao people (mainly Vietnamese and Chinese) live in the central zone of Vientiane.

**Figure 4:**
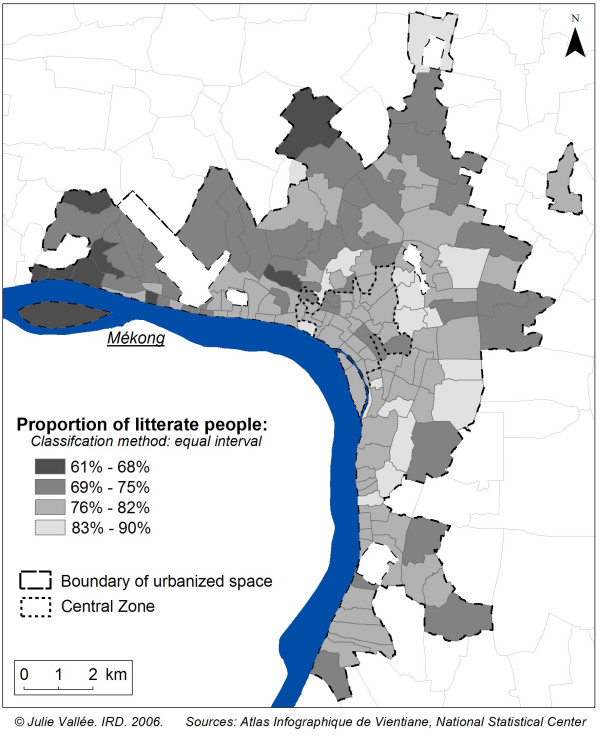
**Spatial distribution of proportion of literate people in 1995**. Spatial distribution of literate people is particularly heterogeneous in the first and second belt of urbanization.

Among every available variable in the 1995 census, we selected two well-known health determinants with unequal spatial distribution within the city proper: nationality (proportion of people who do not have Lao nationality) and the level of education (proportion of people who were literate, in any language). The spatial heterogeneity of these two variables in Vientiane could influence spatial health distribution (Figures 3 and 4). As spatial distribution of non-Lao people (mainly Vietnamese and Chinese) was particularly heterogeneous in the central zone, it was important to control the choice of nine villages to ensure that we did not only select villages with a higher proportion of foreigners than in the central zone. Equally, given that the spatial distribution of literacy was particularly heterogeneous in the first and second belt of urbanisation, we wished also to ensure that the selected villages would not be so alike in terms of literacy.

**Figure 5:**
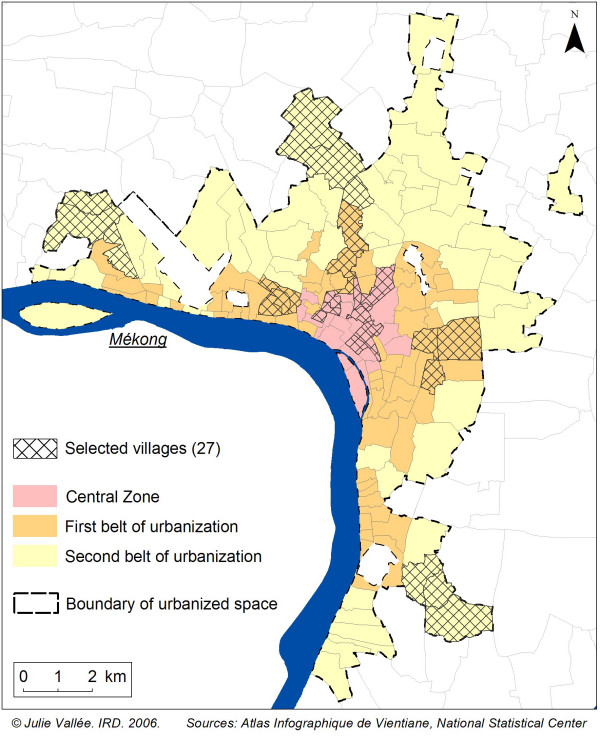
**27 selected villages for health survey**. In every selected village (clustered), around 74 adults and 74 children were planned to be interviewed.

As described earlier, we calculated, for every combination of three groups of three adjacent villages, the mean squared difference of the proportion of those with Lao nationality and compared it with the variance of the proportion of those with Lao nationality calculated at the stratum level. We followed the same steps with the proportion of literacy. After this two-step procedure, we obtained a few combinations in each stratum whose variability of nationality and education was very similar to the variability calculated at the stratum level. We chose from among these the combination villages for which there were no logistical problems (such as requiring political authorisation to carry out research there). This procedure provided three combinations of nine villages (Figure 5). Table 2 summarises the characteristics (obtained from the 1995 census) of these three combinations in comparison with the corresponding urban stratum. As a rough guide, 1928 people lived on average in every 27 selected villages, with a minimum of 562 and a maximum of 4513 inhabitants per village (2005).

**Table 2:** Characteristics (in 1995 census) of selected combinations in comparison with the corresponding urban stratum

		Lao nationality		Literate people	
		
		Proportion (%)	Variance*	Proportion (%)	Variance*
Central zone	27 villages	89.9	0.00424	77.7	0.00118
	9 selected villages	89.4	0.00441	76.4	0.00117
First urbanised belt	67 villages	98.3	0.00050	79.1	0.00198
	9 selected villages	98.1	0.00040	77.4	0.00196
Second urbanised belt	56 villages	99.5	0.00012	73.8	0.00325
	9 selected villages	99.7	0.00001	70.5	0.00341

* For 9 selected villages, variance corresponds to the mean difference (high squared) between the proportion in every 9 villages and the mean proportion calculated at the layer level.

### Random selection of respondents in every selected village

To obtain a sample in every selected village as reliably as possible (not only statistically but also spatially) respondents needed to be selected randomly. With the help of village authorities, we created a sampling frame in each village and then selected households to survey at random. Within each selected household, a maximum of one adult and one child were allowed to participate in the survey. The probability of household selection was proportional to the number of eligible people in the household.

## Discussion

In developing this sampling frame, the main difficulty we encountered was the lack of data available on the population. We needed to ascertain accurate information about some health determinants and about their spatial repartition. For the Vientiane survey, only urban data from 1999 with aerial photographs, demographic data from the 1995 census, and the number of inhabitants per village from 2005 census were available. Precise demographic data from the 2005 census (such as literacy, nationality, access to electricity, water, and latrine access) were not yet available during the preparation for this survey.

For the health survey in Vientiane, we adopted a two-stage selection procedure with a first non-random stage of selection of clusters: we chose clusters that would be representative of the urban and health variability of the global population. Conventional random cluster sampling is certainly statistically appropriate when the number of clusters is large. However, when only a small number of clusters are sampled in order to correspond to geographic objectives and/or to logistical needs, it becomes statistically more appropriate to choose clusters instead of randomly select them. Where this method is used, the choice of clusters should be based on reasoned hypotheses and on the specific research objectives. A modified clustered design with a first non-random stage of cluster selection can provide appropriate information both to study health spatial interactions and to estimate other health variables, such as prevalence, at the city level.

## Competing interests

The author(s) declare that they have no competing interests.

## Authors' contributions

JV performed the sample design, organized the Vientiane health survey and drafted the manuscript; MS and FF took part in the reasoning associated with the construction of the survey sample and corrected the manuscript. MS contributed also to the conception of the sample design; AB, VM made comments about geographical approach and organized Vientiane health survey; KP and GS participated to survey organization. All authors read and approved the final manuscript.

## References

[B1] Tobler WR (1970). A Computer Model Simulating Urban Growth in the Detroit Region. Economic Geography.

[B2] Waller L, Gotway C (2004). Applied Spatial Statistics for Public Health Data.

[B3] Diez Roux A (2001). Investigating Neighborhood and Area Effects on Health. American Journal of Public Health.

[B4] (2003). Asian Development Bank (ADB) & MCTPC, Dept of Housing and Urban Planning. Lao Urban Data Book, Development Indicators for the Urban Areas of Lao PDR, Draft Final report.

[B5] Cochran WG (1977). Sampling Techniques.

[B6] Curtis S (2004). Health and Inequality. Geographical perspectives.

[B7] Hendersen RH, Sundarset T (1982). Clustered sampling to assess immunization coverage: a review experience with a simplified method. Bull WHO.

[B8] Gould P (1970). Is statistic inferens the geographical name for a wild goose?. Economic geography.

[B9] Zelin A, Stubbs R (2005). Clustered Sampling: A False Economy?. International Journal of Market Research.

[B10] Vallée J (2007). Espace urbanisé et périmètres urbains, une délimitation complexe. Vientiane, développement urbain et patrimoine, Keophilavan Aphaylat, Pierre Clément, Charles Goldblum et Christian Taillard (édit), les Cahiers de l'Ipraus, Editions Recherches, Paris, forthcoming in.

[B11] Rossi G, Tissandier P, Inthiphone B (2003). Atlas infographique de Vientiane, Vientiane, Programme de formation-recherche en coopération inter-universitaire, Université Nationale du Laos, Université de Bordeaux III.

